# Prostate Cancer Screening Among American Indians and Alaska Natives: The Health and Retirement Survey, 1996–2008

**DOI:** 10.5888/pcd12.150088

**Published:** 2015-08-06

**Authors:** R. Turner Goins, Marc B. Schure, Carolyn Noonan, Dedra Buchwald

**Affiliations:** Author Affiliations: Marc B. Schure, Health Services Research and Development, VA Puget Sound Health Care System, Seattle, Washington; Carolyn Noonan, Dedra Buchwald, Department of Epidemiology, School of Public Health, University of Washington, Seattle, Washington.

## Abstract

**Introduction:**

Among US men, prostate cancer is the leading malignancy diagnosed and the second leading cause of cancer death. Disparities in cancer screening rates exist between American Indians/Alaska Natives and other racial/ethnic groups. Our study objectives were to examine prostate screening at 5 time points over a 12-year period among American Indian/Alaska Native men aged 50 to 75 years, and to compare their screening rates to African American men and white men in the same age group.

**Methods:**

We analyzed Health and Retirement Study data for 1996, 1998, 2000, 2004, and 2008. Prostate screening was measured by self-report of receipt of a prostate examination within the previous 2 years. Age-adjusted prevalence was estimated for each year. We used regression with generalized estimating equations to compare prostate screening prevalence by year and race.

**Results:**

Our analytic sample included 119 American Indian/Alaska Native men (n = 333 observations), 1,359 African American men (n = 3,704 observations), and 8,226 white men (n = 24,292 observations). From 1996 to 2008, prostate screening rates changed for each group: from 57.0% to 55.7% among American Indians/Alaska Natives, from 62.0% to 71.2% among African Americans, and from 68.6% to 71.3% among whites. Although the disparity between whites and African Americans shrank over time, it was virtually unchanged between whites and American Indians/Alaska Natives.

**Conclusion:**

As of 2008, American Indians/Alaska Natives were less likely than African Americans and whites to report a prostate examination within the previous 2 years. Prevalence trends indicated a modest increase in prostate cancer screening among African Americans and whites, while rates remained substantially lower for American Indians/Alaska Natives.

## Introduction

In US men, prostate cancer is the leading malignancy diagnosed and the second leading cause of cancer death (1–3). Prostate cancer affects approximately 144 per 100,000 men aged 50 to 54 years; this number increases rapidly with age to 930 per 100,000 men aged 70 to 74 years. Age-adjusted mortality rates predict that 23.6 of 100,000 men will die each year from complications resulting from prostate cancer (1).

The primary goal of prostate screening (eg, prostate-specific antigen [PSA] test, digital rectal examination) is to identify men for whom treatment would reduce morbidity and mortality. Guidelines for prostate screening differ across medical organizations (4); recently, the US Preventive Services Task Force (USPSTF) changed its guidelines. In 2008, USPSTF concluded that evidence was insufficient to assess the balance of benefits and harms of screening for prostate cancer in men aged younger than 75 years and recommended against screening for prostate cancer in men aged 75 years or older (5). In 2012, USPSTF recommended against PSA-based screening for average-risk men of all ages (6). The American Urological Association recently recommended shared decision making for men aged 55 years or older regardless of risk (7). The American Cancer Society recommends that men make informed decisions by considering the available information, having a discussion with their physician, and exploring their own views on the benefits and side effects of screening and treatment (8). The rationale against testing for prostate cancer is that the cancer is often slow-growing and may never progress enough to threaten one’s life. Thus, since treatment carries risks, finding and treating early tumors may do more harm than good for some patients. Although stakeholders have not reached consensus on prostate screening recommendations, screening can provide an opportunity for early detection, when cancer treatment is most effective (9,10).

Relatively little is known about prostate cancer and screening behaviors among American Indian and Alaska Native (AI/AN) men. Some studies indicate that AI/AN men have a lower incidence of prostate cancer than white men (11,12), yet AI/ANs have the highest risk of prostate cancer mortality of any racial group (11,13). A recent examination of prostate cancer mortality found that rates were higher for AI/ANs than for whites and that mortality declined from 1999 to 2009 for whites but not for AI/ANs (11). Another recent study found that the prostate cancer mortality-to-incidence ratio from 1999 to 2009 was higher among AI/ANs than among whites (14).

An examination of the 637 Indian Health Service Contract Health Services Delivery Area counties found lower age-adjusted prostate cancer incidence rates among AI/AN men than among white men in all regions except the Southern Plains. Rates of age-adjusted prostate cancer incidence among AI/AN men ranged from 81.3 in the Southwest to 164.1 in the Northern Plains. This range was smaller among white men, ranging from 132.1 in the Southwest to 165.4 in Alaska. The same study found higher rates of age-adjusted prostate cancer death in the Northern and Southern Plains and a lower death rate in the East among AI/AN men than among white men. Prostate cancer death rates among AI/AN men ranged from 22.4 in the Southwest to 41.2 in the Northern Plains and among white men, rates ranged from 23.4 in the East to 26.9 in the Pacific Coast (11).

Accurate estimates of prostate screening rates among AI/ANs are challenged by small, nonrepresentative samples and widespread racial misclassification in vital statistics and cancer registry data. Nonetheless, several studies suggest that AI/AN men have lower prostate screening rates than men of other races and ethnicities (12,15–17). Three of the 4 studies that compared prostate screening rates between AI/AN men and men of other races and ethnicities combined data from multiple years of the Behavioral Risk Factor Surveillance System (12,15,17), and the fourth study used the 2001 California Health Interview Survey (16).

Given the limited available research literature, we set out to examine prostate screening at 5 time points during a 12-year period among AI/AN men aged 50 to 75 years and to compare their screening rates with those of African American men and white men in the same age range.

## Methods

### Data sources

The Health and Retirement Study (HRS) is a national panel survey of US adults aged 50 years or older and their spouses. Its aim is to obtain information on labor force participation and health (18). The HRS uses a national area probability sample of US households with supplemental oversamples of African Americans and Hispanics, but not of AI/ANs (19). Every 2 years, the HRS conducts core interviews either via telephone or in person. An in-person interview is the primary mode used for baseline interviews and for those aged 80 years or older. From the beginning of the HRS in 1992 to 2002, the data collection method for follow-up interviews with sample members younger than 80 was telephone. A larger number of interviews were done in person in 2004, and since 2006 half of the sample is assigned to an in-person interview (20). Data from multiple years for the same participant are linked via a unique identification number.

We selected the HRS for this study because our research focus is on older AI/AN men and this data set has a rich set of longitudinal data on a relatively large number of older AI/AN men that are available through only a few data sets (21). We used HRS data collected in 1996 (Wave 3), 1998 (Wave 4), 2000 (Wave 5), 2004 (Wave 7), and 2008 (Wave 9). We omitted 2002 (Wave 6) and 2006 (Wave 8) because they did not collect data on prostate screening (18). Men who responded to an item on prostate screening at least once during these 5 waves were included in our analyses.

### Measures

We used self-reported data on prostate screening, consisting of responses to the question “In the past two years, have you had an examination of your prostate to screen for cancer?” This is the only prostate screening-related question in the HRS, and this item was consistently worded across all waves included in our study. Age and race were also self-reported.

### Statistical analysis

We used an analysis of variance model to describe and compare age according to race. For each wave, we used Poisson regression to compute age-adjusted prevalence of prostate screening for each racial group. Wave-specific sampling weights were used to adjust for the complex survey design of the HRS. The trend in prevalence of prostate screening over time for each racial group was examined by using a generalized estimating equations regression that allows for all available data for each participant, even if data on participants were missing from one or more waves (22). Models were fit by using a Poisson distribution and log link to estimate relative risk (RR), because odds ratios from logistic regression may be biased away from the null when the outcome is not rare (23,24). We were most interested in the main effect of race (3 levels: AI/AN, African American, and white), obtained by comparing the average prevalence of prostate screening across all data waves for participants in these categories. We also examined the main effect of time (ie, wave at 5 levels) and the interaction of time and race to determine the significance of differences in prevalence of screening over time and assess whether the pattern of change over time was comparable in all 3 groups. The regression analyses adjusted for age. Analyses were conducted with Stata SE 12.1 for Windows (25).

## Results

Across all 5 waves of HRS data, data on 9,704 men were available for analyses, including 119 AI/ANs (n = 333 observations), 1,359 African Americans (n = 3,704 observations), and 8,226 whites (n = 24,292 observations) (Table 1). About half (45%) of participants had data from 4 waves, 15% from 3 waves, 25% from 2 waves, and 15% from 1 wave. Only 1 man had data from all 5 waves. Age at the first wave (Wave 3 in 1996) differed significantly according to race, but the differences were not large, with a mean (standard deviation) age of 59.2 (7.7) years for AI/ANs, 60.0 (7.3) years for African Americans, and 61.0 (7.7) years for whites.

**Table 1:** Unweighted Number of Male Participants by Year and Race, Health and Retirement Study, 1996–2008

Year	American Indian/Alaska Native	African American	White
1996	64	776	4,856
1998	21	245	1,868
2000	79	866	5,931
2004	94	994	6,290
2008	75	823	5,347
Total no. of observations	333	3,704	24,292
No. of men available for analysis^a^	119	1,359	8,226

a About half (45%) of participants had data from 4 waves, 15% from 3 waves, 25% from 2 waves, and 15% from 1 wave. Only 1 man had data from all 5 waves.

From 1996 to 2008, prostate screening rates changed for each group: from 57.0% to 55.7% among American Indians/Alaska Natives, from 62.0% to 71.2% among African Americans, and from 68.6% to 71.3% among whites. (Table 2). Averaged across all waves, the age-adjusted likelihood of prostate screening was significantly lower among AI/ANs than among African Americans (RR = 0.81; 95% confidence interval [CI], 0.71–0.92) and whites (RR = 0.79; 95% CI, 0.70–0.90).

**Table 2:** Weighted Age-Adjusted Prevalence of Prostate Screening by Year and Race, Health and Retirement Study, 1996–2008

Year	American Indian/Alaska Native, % (95% Confidence Interval)	African American, % (95% Confidence Interval)	White, % (95% Confidence Interval)
1996	57.0 (38.4–75.6)	62.0 (57.2–66.9)	68.6 (66.7–70.5)
1998	36.1 (7.9–64.2)	70.8 (65.6–75.9)	67.3 (64.3–70.2)
2000	55.6 (41.2–69.9)	73.1 (68.5–77.7)	73.2 (71.7–74.8)
2004	60.0 (45.8–74.2)	71.2 (67.9–74.5)	73.3 (71.7–75.0)
2008	55.7 (39.2–72.3)	71.2 (69.2–77.4)	71.3 (69.5–73.2)

We found a significant time-by-race interaction (*P* = .01). In an examination of the main effect of time separately in each racial group, we observed a trend for increased prevalence of prostate screening with time in all 3 racial groups. However, the trend was not significant among AI/AN men (RR = 1.03; 95% CI, 0.98–1.07); but it was significant for African American men (RR = 1.03; 95% CI, 1.02–1.04) and white men (RR = 1.02; 95% CI, 1.01–1.02).

## Discussion

This nationally representative, population-based study of older men demonstrates that prostate screening rates in recent decades were markedly lower among AI/AN men than among African American and white men. From 1996 to 2008, rates of prostate screening within the previous 2 years were consistently 60.0% or below among AI/ANs aged 50 to 75 years, whereas they ranged from 62.0% to 73.1% among African Americans and from 68.6% to 73.3% among whites. During the 12-year period, screening rates showed a pattern of significant increase among whites and African Americans but not among AI/ANs. The disparity in screening between AI/AN men and white men was constant, while the disparity between AI/AN men and African American men increased. Our study suggests that there has been no meaningful improvement in closing the prostate screening gap between AI/AN men and their African American and white counterparts.

Our study is one of several studies to report disparities in cancer screening rates between AI/ANs and other racial/ethnic groups. Screening disparities have been reported for breast cancer, cervical cancer, and colorectal cancer (15–17). AI/ANs lag behind their same-age counterparts in all other racial groups for achieving Healthy People 2020 targets for almost all common cancer screenings (26). Consistent with previous findings of screening disparities, our findings show that AI/ANs had the lowest prostate screening rates of the 3 racial groups evaluated (12,15–17). Researchers continue to speculate on the possible reasons for these persistent disparities; recent studies have identified low socioeconomic status (15), lack of cancer care services (27,28), language differences (27), illness beliefs (27,29), limited knowledge of cancer care (27,29,30), negative attitudes toward cancer treatment (30), transportation difficulties (27), and perceived discrimination by the health care provider (31) as potential contributing factors.

Our results should be interpreted in the context of several limitations. First, our data on prostate screening were based on self-report and therefore may be subject to recall bias. Such bias might over- or underestimate screening prevalence. Second, our dependent variable was based on whether respondents received any kind of prostate examination, so we cannot make direct comparisons with other prostate screening studies on the basis of data that specified a type of screening test. Similarly, the time frame of reference in the HRS for receiving an examination was 2 years, so we cannot directly compare screening prevalence in our study with that of other national surveys that use a different time frame, such as the National Health Interview Survey. Third, although the overall HRS sample was large, the HRS does not oversample AI/ANs. Consequently, subgroup analyses included a much smaller sample of AI/AN men than men of other races. This smaller sample size of AI/AN men makes the results for AI/ANs less statistically reliable than those for African American men and white men, and our results should be carefully interpreted. Also, this small sample size prevents us from examining the regional differences in prostate screening receipt reported in previous work (11,12,15,17). Directions for future research should include improved surveillance methods and survey methodology for AI/ANs.

For AI/AN men, programs and policies will probably need to be tailored, because AI/AN populations are culturally diverse and face a wide range of barriers to health care access. From 2005 to 2015, the National Cancer Institute sponsored the Community Networks Program Centers in an effort to reduce cancer health disparities through community-based participatory engaged education, training, and research among underserved populations. This initiative was responsible for 3 centers that developed and implemented culturally and regionally tailored programs to reduce cancer disparities among AI/ANs.

In addition, policies are needed to address access barriers, particularly barriers to age-appropriate health screening. For example, urban AI/ANs have more limited access to Indian Health Services than AI/ANs who reside on tribal lands (32). Similarly, older adults may require additional support mechanisms, such as transportation, to facilitate access to health care facilities and other preventive services. Future research should assess regional differences as well as cultural barriers and facilitators to explain disparities in prostate cancer screening across racial groups.
